# Label-Free Detection of Human Glycoprotein (CgA) Using an Extended-Gated Organic Transistor-Based Immunosensor

**DOI:** 10.3390/s16122033

**Published:** 2016-11-30

**Authors:** Tsukuru Minamiki, Tsuyoshi Minami, Yui Sasaki, Shin-ichi Wakida, Ryoji Kurita, Osamu Niwa, Shizuo Tokito

**Affiliations:** 1 Research Center for Organic Electronics, Graduate School of Science and Engineering, Yamagata University, 4-3-16 Jonan, Yonezawa, Yamagata 992-8510, Japan; tey14898@st.yamagata-u.ac.jp (T.M.); txe37183@st.yamagata-u.ac.jp (Y.S.); tokito@yz.yamagata-u.ac.jp (S.T.); 2 Japan Society for the Promotion of Science (JSPS), Ichibancho, Chiyoda-ku, Tokyo 102-8471, Japan; 3 Institute of Industrial Science, The University of Tokyo, 4-6-1 Komaba, Meguro-ku, Tokyo 153-8505, Japan; 4 Biomedical Research Institute, National Institute of Advanced Industrial Science and Technology (AIST), 1-8-31 Midorigaoka, Ikeda, Osaka 563-8577, Japan; s.wakida@aist.go.jp; 5 Biomedical Research Institute, National Institute of Advanced Industrial Science and Technology (AIST), Tsukuba Central 6, 1-1-1 Higashi, Tsukuba, Ibaraki 305-8566, Japan; r.kurita@aist.go.jp; 6 Advanced Science Research Laboratory, Saitama Institute of Technology, Fukaya, Saitama 369-0293, Japan; niwa@sit.ac.jp

**Keywords:** chromogranin A, glycoprotein, organic field-effect transistor, immunosensor, extended-gate, label-free, antibody immobilization, self-assembled monolayer

## Abstract

Herein, we report on the fabrication of an extended-gated organic field-effect transistor (OFET)-based immunosensor and its application in the detection of human chromogranin A (hCgA). The fabricated OFET device possesses an extended-gate electrode immobilized with an anti-CgA antibody. The titration results of hCgA showed that the electrical changes in the OFET characteristics corresponded to the glycoprotein recognition ability of the monoclonal antibody (anti-CgA). The observed sensitivity (detection limit: 0.11 µg/mL) and selectivity indicate that the OFET-based immunosensor can be potentially applied to the rapid detection of the glycoprotein concentration without any labeling.

## 1. Introduction

Chromogranin A (CgA), which is one of the glycoproteins, exists in secretory granules [1]. CgA is processed into small peptides, several of which affect the secretory function of the parent cells [2]. CgA is co-stored and co-released with catecholamines from storage granules in the adrenal medulla, or with the parathyroid gland [2]. Serum CgA is generally applied as an early biomarker for monitoring in several diseases, such as endocrine tumors, heart failure, hypertension, and neurodegenerative/neuropsychiatric diseases [3,4,5,6]. In addition, salivary CgA is also known as a marker of psychophysical stress [7,8], such as exposure to situations of anxiety [9,10,11] and depressive mood [12,13]. For these reasons, the monitoring of CgA levels is very important for the management of our health conditions. 

In general, immunoassays, such as radioimmunoassays (RIAs), enzyme-linked immunosorbent assays (ELISAs), fluorescent and chemiluminescent immunoassays, have been developed for protein determination [14]. Although such immunoassays have high selectivity and sensitivity, they are relatively complicated due to the necessity of labels and relatively large-sized equipment. Therefore, these methods are not amenable to easy and low-cost testing of glycoproteins. Among biosensor platforms such as quartz crystal microbalance (QCM), field-effect transistor (FET) and surface plasmon resonance (SPR), FET-based sensors are one of the promising approaches owing to their simplicity and capability of highly sensitive detection based on portable/compact sensor systems. Accordingly, carbon nanomaterial field-effect transistor (FET)-based biosensors for the detection of CgA were preliminary studied [15]. However, the FET-based biosensors for CgA detection are in their early stages.

Organic field-effect transistors (OFETs) are one of the best candidates for chemical/biosensor devices because of their low-cost processability and mechanical flexibility [16]. The development of OFET-based biosensors has just begun to bloom in recent years. For example, OFET-based sensors have been applied to the detection of biologically active substances such as proteins [17,18,19,20], saccharides [21], etc. Especially, we have developed an OFET-based immunoassay to detect immunoglobulin [19,20]. Toward that end, we herein demonstrate the label-free immunosensor device based on an OFET for glycoproteins such as human CgA (hCgA).

## 2. Materials and Methods

The OFET-based immunosensor was composed of two components; the stable operation of the fabricated device was achieved by isolating of the drive unit (the OFET) and the detection portion (the extended-gate electrode). The details of the device fabrication were described in our previous report [22].

**Figure 1:**
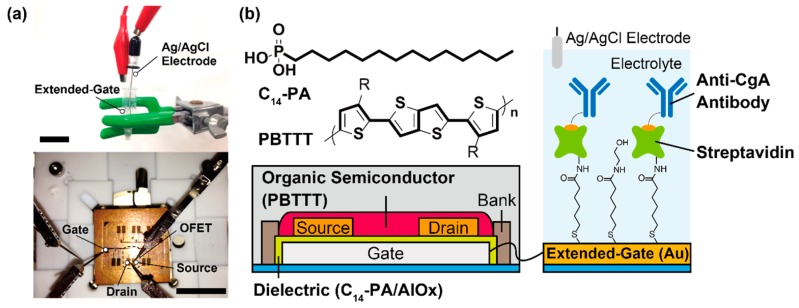
(**a**) Photograph of the fabricated device (above: the detection portion, below: the OFET). The scale bars are 2 cm; (**b**) Schematic illustration of the OFET-based immunosensor.

PBTTT (poly{2,5-bis(3-hexadecylthiophene-2-yl)thieno[3,2-*b*]thiophene} [23])-based FET with the tetradecylphosphonic acid (C_14_-PA)/AlOx dielectric layer [24] was designed for an accurate electrical detection of CgA under ambient conditions (Figure 1). All electrodes were deposited on a substrate via thermal vacuum evaporation (30 nm in thickness). To improve the device stability, the OFET part was fully covered with an amorphous fluorinated polymer (Cytop^®^ CTL-809M, Asahi Glass, Tokyo, Japan) by spin-coating. Additionally, an extended-gate electrode consists of the deposited Au (50 nm in thickness) modified with a self-assembled monolayer (SAM) of 5-carboxy-1-pentanethiol (CPT, Dojindo Laboratories, Kumamoto, Japan).

For the label-free detection of hCgA, the modification scheme of the extended-gate electrode is described as follows. First, a MES (2-morpholinoethanesulfonic acid)-buffer solution (100 mM, pH 5.5) containing *N*-hydroxysulfosuccinimide (sulfo-NHS, 5 mM, Thermo Fisher Scientific Inc., Waltham, MA, USA), *N*,*N*’-diisopropylcarbodiimide (*N*,*N*’-DIC, 40 mM, Kanto Kagaku, Tokyo, Japan) was dropped onto the Au electrode modified with the CPT-SAM for 15 min at room temperature. Then, streptavidin (500 µg/mL, Wako, Osaka, Japan) solved in a carbonate buffer (Na_2_CO_3_: 15 mM, NaHCO_3_: 35 mM, pH 9.6) was casted onto the electrode to incubate for 2 h at room temperature. After that, 2-aminoethanol (1 M, Tokyo Kasei, Tokyo, Japan) in a PBS (phosphate buffer saline) solution (KCl: 2.7 mM, NaCl: 136 mM, KH_2_PO_4_: 1.5 mM, Na_2_HPO_4_: 8.1 mM) was dropped onto the electrode for 15 min at room temperature. To block a non-specific adsorption of proteins onto the electrode, the electrode was immersed in a PBS solution with Tween 20 (0.05 wt %, purchased from Kanto Kagaku, Tokyo, Japan) and human serum albumin (HSA, 0.1 wt %, purchased from Wako, Osaka, Japan) for 15 min. Then, the electrode was immersed in a PBS solution of biotin-tagged anti-CgA antibody (30 µg/mL) with 0.1 wt % HSA, and allowed to incubate for 30 min at room temperature. The electrode functionalized with anti-CgA antibody (clone code: LK2H10, purchased from Abcam, Cambridge, UK) was immersed in a PBS solution of hCgA (0–50 µg/mL) with HSA (0.1 wt %), or interfering proteins (amylase, immunoglobulin A (IgA), myeloperoxidase) for 15 min at 37 °C. Finally, the analyte concentration was electrically detected by the fabricated OFET. The Ag/AgCl electrode (BAS Inc., Tokyo, Japan) was used as the reference electrode.

## 3. Results and Discussion

### 3.1. Characterization of the Fabricated Device

In a previous report, we characterized the modification of the Au extended-gate electrode using various techniques [19]. We also measured the electrical characteristics of the OFET device. The fabricated OFET can be operated under low voltages (<3 V) [22], indicating that the device can be applied to immunosensing in aqueous media.

### 3.2. Label-Free Electrical Detection of hCgA

After immobilizing the biotin-tagged anti-CgA antibody on the extended-gate electrode, we immersed the electrode into the PBS solution with the hCgA, and then we measured the electrical characteristics of the connected OFET. The electrical characteristics of the fabricated OFET in titration experiments were measured by a source meter (2636B, Keithley, Cleveland, OH, USA). To estimate the output signal (i.e., the threshold voltage, *V*_TH_) from transfer characteristics, the following equation [25] was used:

*I*_DS_ = (*W*/2*L*)*μC*(*V*_GS_–*V*_TH_)^2^, (1)

where *I*_DS_ is the drain current, *W* and *L* are the channel width (1000 μm) and length (50 μm), μ is the field-effect mobility, *C* is the capacitance of the gate dielectric (~0.8 μF/cm^2^) [26], and *V*_GS_ is the gate voltage. In addition, the electrical response (Δ*V*_TH_) induced by the charged analyte (the charge density, *Q*) can be expressed in the following equation:

Δ*V*_TH_ = Δ*Q*/*C,* (2)

meaning that the shift of the transfer characteristics can be affected by the charge where the captured analyte is bound on the extended-gate electrode [27,28].

**Figure 2:**
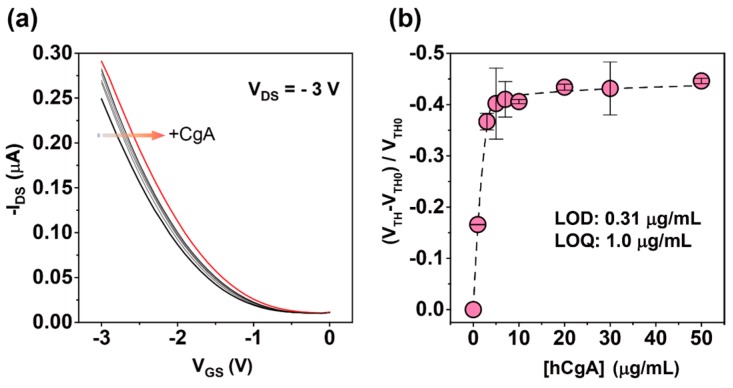
(**a**) Transfer characteristics (*I*_DS_-*V*_GS_) of the OFET upon titration with hCgA in a PBS solution with 0.1 wt % HSA. [Human CgA] = 0, 1, 3, 5, 7, 10, 20, 30, and 50 µg/mL; (**b**) Changes in the threshold voltage (*V*_TH_) of the OFET by hCgA at various concentrations in a PBS solution with 0.1 wt % HSA. Five repetitions were measured for each concentration.

Figure 2a shows that the transfer characteristics of the OFET device were positively shifted upon the addition of the increased concentration of the hCgA. The reason for the positive shift of the extended-gated FET is generally explained by the interfacial potential shift at the extended-gate/electrolyte interface [29]; these results suggest that the negatively charged hCgA was captured on the extended-gate electrode [30]. In Figure 2b, we plotted the relationship between the concentration of the hCgA and the electrical responses of the OFET device (changes in the threshold voltage). Because an extended-gated FET is one of the potentiometric-based electrochemical sensors [31], the threshold voltage is directly reflected in the interfacial potential shift at the extended-gate/electrolyte interface. 

The addition of hCgA over 5.0 µg/mL induced a saturated response, most likely due to the anti-CgA being fully bound to the hCgA (Figure 2b). The limit of detection (LOD) [32] and the limit of quantification (LOQ) [32] were estimated to be 0.31 µg/mL (~6 nM) and 1.0 µg/mL (~19 nM), respectively, meaning that the sensitivity was comparable to that of a carbon nanotube (CNT)-based FET sensor device for CgA [15]. More importantly, the fabrication process for our designed OFET was much simpler than that for the photolithographic technology-based CNT-FET (see Materials and Methods section). Although the sensitivity for the hCgA in conventional immunoassays (e.g., ELISA) was higher than our fabricated immunosensor [33], the protocol for the label-free detection based on the OFET is much simpler. Additionally, the assay time of the fabricated OFET immunosensor (~0.5 h) is quicker than an ELISA method (~2.5 h) [34]. Furthermore, the sensitivity of our fabricated OFET could be improved by connecting the device with OFET-based amplifier circuits [35].

### 3.3. Selectivity

**Figure 3:**
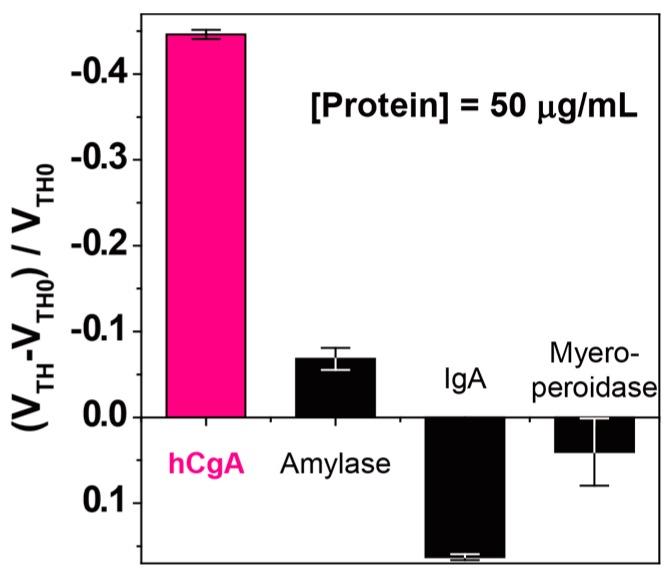
Changes in the threshold voltage (*V*_TH_) of the OFET by the proteins at various concentrations in a PBS solution with 0.1 wt % HSA. [Protein] = 50 µg/mL. Five repetitions were measured for each analyte.

To investigate the selectivity of the fabricated OFET immunosensor, we also measured various analytes using the same device. We chose IgA [36], amylase [37,38], and myeloperoxidase [39] as the analytes (they are regularly contained in human saliva). As a result, a selective response to the hCgA was observed (Figure 3). Because of its relatively large protein size, the observed weak response to the addition of IgA might be derived from a physical adsorption onto the extended-gate electrode. The utilized monoclonal antibody (clone code: LK2H10) has high specificity for the hCgA [40], meaning that the response to hCgA is derived from the immune interaction between the hCgA and the extended-gate electrode immobilized with the anti-CgA antibody. 

### 3.4. CgA Detection in Artificial Saliva

**Figure 4:**
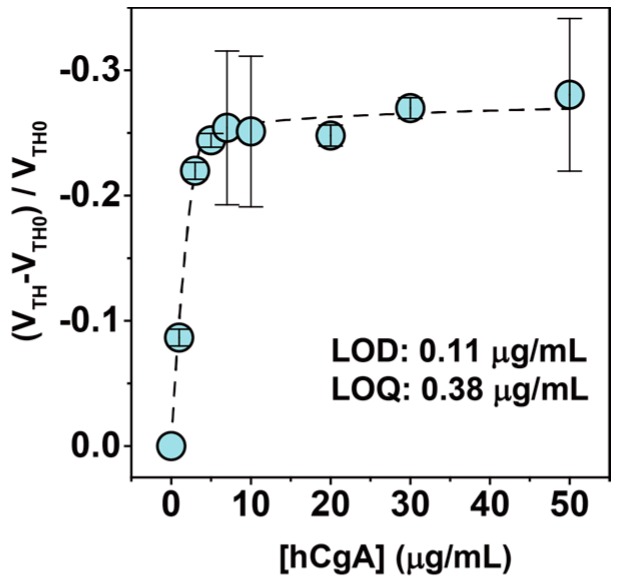
Changes in the threshold voltage (*V*_TH_) of the OFET by the hCgA at various concentrations in Saliveht^®^ which contains NaCl, KCl, CaCl_2_, MgCl_2_, and K_2_HPO_4_. [Human CgA] = 0–50 µg/mL. Five repetitions were measured for each concentration.

Based on previous reports [7,8], the CgA level in human saliva might be applied as a psychological stress marker. Therefore, for evaluation of the feasibility of the OFET-based immunosensor in practical applications, we finally attempted the detection of hCgA in a commercially available artificial saliva (Saliveht^®^ Aerosol, purchased from Teijin Pharma Co. Ltd., Tokyo, Japan) containing many chemical species (NaCl, KCl, CaCl_2_, MgCl_2_, and K_2_HPO_4_) for medication. As a result, the threshold voltage of the fabricated OFET device was positively shifted by increasing the hCgA level (Figure 4), suggesting that the fabricated OFET could be used for the selective detection of the hCgA contained in biological fluids such as saliva. The observed response denotes a similar tendency as the titration experiment in a PBS solution (Figure 2). In an artificial saliva experiment, the LOD and the LOQ were estimated to be 0.11 µg/mL (~2 nM) and 0.38 µg/mL (~7 nM), respectively.

## 4. Conclusions

In conclusion, we have successfully demonstrated an electrical label-free immunosensing of hCgA using an extended-gated OFET device immobilized with anti-CgA antibody. The detection limit (0.11 µg/mL in the artificial saliva) was comparable to that of the reported FET-based sensor [15]. Although the estimated LOD value (~2 nM) is seven times higher than the physiological level of hCgA (~0.3 nM [41]), the sensitivity could be improved by FET-based electrical circuits [35,42]. Additionally, our fabricated device is much simpler and more rapid than the conventional immunoassay (e.g., ELISA) for hCgA detection (the assay time is ~30 min). In previous research, we succeeded in decreasing the assay time (~1 min) for a phosphoprotein using by an OFET sensor functionalized with an “artificial receptor” [43]. Therefore, further improvement of the assay time for hCgA could be achieved with similar approaches (i.e., an OFET sensor functionalized with an artificial receptor for hCgA). Hence, we believe that our preliminary results on the OFET-based immunosensors could pave the way for a new approach in the monitoring of hCgA levels for human health conditions in daily life.
